# Antibacterial activity and mechanism of naphthoquine phosphate against ceftazidime-resistant *Acinetobacter baumannii* via cell membrane disruption and ROS induction

**DOI:** 10.3389/fmicb.2025.1603462

**Published:** 2025-08-04

**Authors:** Yongtian Yuan, Liangliang Zhao, Zhuchun Bei, Baogang Wang, Dongna Zhang, Likun Xu, Jiahui Liu, Meng Lv, Qin Xu, Yabin Song

**Affiliations:** ^1^Artemisinin Research Center, Guangzhou University of Chinese Medicine, Guangzhou, China; ^2^State Key Laboratory of Pathogen and Biosecurity, Academy of Military Medical Sciences, Beijing, China

**Keywords:** naphthoquine phosphate, *Acinetobacter baumannii*, antibacterial activity, cell membrane permeability, transcriptome analysis

## Abstract

**Introduction:**

Drug-resistant bacteria, particularly *Acinetobacter baumannii*, present a significant threat to global public health, highlighting the urgent need for novel antibacterial therapies. Drug repurposing has emerged as a promising strategy to accelerate therapeutic development by identifying new applications for existing pharmaceuticals. This study investigates the potential of naphthoquine phosphate (NQP), an antimalarial agent, as a broad-spectrum antibacterial candidate against the multidrug-resistant strain *A. baumannii* LAC-4.

**Methods:**

To evaluate the antibacterial activity of NQP, we determined the minimum inhibitory concentration (MIC) against *Acinetobacter baumannii* LAC-4. Inhibition kinetics were analyzed to assess concentration-dependent effects. Membrane permeability assays were performed to examine NQP-induced changes in cell membrane integrity. Oxidative damage tests were conducted to investigate impacts on bacterial metabolic processes. Morphological changes in *A. baumannii* LAC-4 treated with NQP of MIC were observed using transmission electron microscopy (TEM) and scanning electron microscopy (SEM). Additionally, transcriptome analysis was performed to identify disrupted physiological pathways associated with NQP exposure.

**Results and discussion:**

NQP exhibited broad-spectrum antibacterial activity, with a MIC of 62.5 μg/mL against *Acinetobacter baumannii* LAC-4. Its inhibition kinetics curve confirmed a concentration-dependent inhibitory effect. Membrane permeability tests revealed that NQP disrupts cell membrane integrity, enhancing permeability—consistent with TEM/SEM observations showing significant structural damage in NQP-treated *A. baumannii*, including membrane rupture, cellular deformation, and cytoplasmic disorganization. Oxidative damage tests indicated NQP impacts bacterial metabolism, and transcriptome analysis further demonstrated that NQP disrupts multiple physiological pathways, primarily through enhanced membrane permeability and induced oxidative stress. These findings support NQP as a promising molecular scaffold for developing novel therapies against *Acinetobacter baumannii* infections, highlighting its potential in drug repurposing strategies for combating drug resistance.

## 1 Introduction

Currently, drug-resistant bacteria, notably exemplified by the ESKAPE pathogens, pose a significant threat to global human health (Ho et al., 2025; Puri et al., 2024; Salam et al., 2023). As of 2019, it is estimated that bacterial pathogens with AMR (antimicrobial resistance) have been directly responsible for approximately 1.3 million deaths globally (Antimicrobial Resistance Collaborators, 2022; De Oliveira et al., 2020). The ESKAPE pathogens include *Enterococcus faecium*, *Staphylococcus aureus*, *Klebsiella pneumoniae*, *Acinetobacter baumannii*, *Pseudomonas aeruginosa* and *Enterobacter* spp. (Daruka et al., 2025; Miller and Arias, 2024). The World Health Organization (WHO) has also identified *carbapenem-resistant Enterobacteriaceae (CRE)* and *carbapenem-resistant Acinetobacter baumannii (CRAB)* as the critical priority threats, *carbapenem-resistant Pseudomonas aeruginosa (CRPA)* as the high Priority Bacteria (World Health Organization, 2024).

*Acinetobacter baumannii* (AB) is a well-known Gram-negative, opportunistic pathogen that causes severe infections notoriously difficult to manage and treat, especially in immunocompromised populations such as the elderly, neonates, postoperative patients, and other critically ill individuals (Nocera et al., 2021; Shi et al., 2024). It primarily impacts patients in intensive care units and respiratory medicine departments, with the respiratory system serving as the primary site of infection. Additionally, it has the potential to cause a range of infections, including bacteremia, urinary tract infections, skin and soft tissue infections, and surgical site infections (Cavallo et al., 2023). In the last few years, the annual prevalence of AB has been escalating, earning it recognition as a primary contributor to nosocomial infections on a global scale (Ayoub Moubareck and Hammoudi Halat, 2020; Ma and McClean, 2021). This bacterium, distinguished by its robust biofilm-forming capabilities, demonstrates extraordinary environmental adaptability and multidrug resistance. In recent years, the issue of drug resistance in AB has garnered increasing attention, with numerous reports emerging on the subject (Kyriakidis et al., 2021; Vázquez-López et al., 2020). Initially, AB demonstrated resistance primarily to sulfonamides. However, as antibiotic usage escalated, its resistance spectrum broadened to encompass a wide range of critical antimicrobial agents, including cephalosporins, aminoglycosides, quinolones, and carbapenems (Kyriakidis et al., 2021; Vázquez-López et al., 2020). Clinically, the options for treating AB infections are severely limited, primarily comprising tigecycline, polymyxins, and linezolid (Doi et al., 2015; Vrancianu et al., 2020). Regrettably, over the past decade, strains resistant to these treatments have emerged (Doi et al., 2015; Vrancianu et al., 2020). Strains that exhibit comprehensive resistance profiles present a particularly alarming challenge in clinical therapeutic settings (Luo et al., 2024). Globally, approximately 45% of AB isolates exhibit multidrug resistance (MDR), with the prevalence of MDR AB being four times higher than that of other Gram-negative pathogens, including *Klebsiella pneumoniae* and *Pseudomonas aeruginosa* (Giammanco et al., 2017). These underscore the fact that the drug resistance problem of AB is a continually evolving and increasingly formidable challenge, necessitating the continuous updating of treatment strategies to effectively address the emerging drug resistance phenomenon.

There is an urgent global need for novel antimicrobial therapies, requiring intensified efforts in discovering and developing new drugs. However, the prolonged and challenging process of drug discovery and development exacerbates the difficulty of making new therapies available in a timely manner (Tautermann, 2020). Consequently, there’s an urgent need for strategies to enhance drug development efficiency and expedite the introduction of effective drugs into clinical practice. Drug repurposing, also known as drug repositioning, significantly reduces development time and costs, which is why it’s a popular way to make new medicines (Pushpakom et al., 2019). For example, the combination of fusidic acid and colistin, probably with a third agent (such as minocycline), seems to be the most promising treatment against Carbapenem-Resistant AB (Gontijo et al., 2021; Phee et al., 2015; Phee et al., 2019).

**Figure 1:**
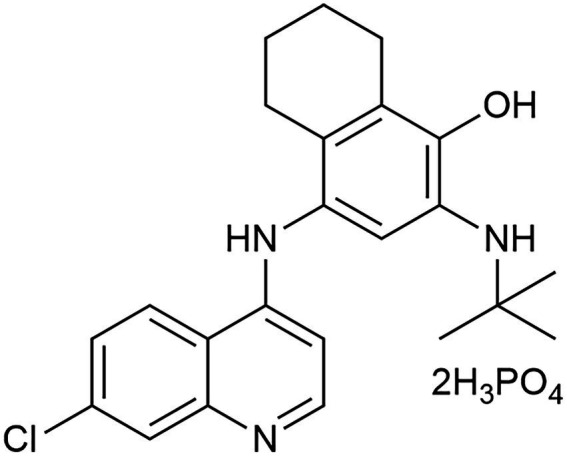
The molecular structure of NQP.

Naphthoquine (NQ), belonging to the quinoline class of antimalarials, is prescribed for the treatment of falciparum malaria, alongside other quinolines such as quinine, chloroquine (CQ), and hydroxychloroquine (HCQ) (Moore et al., 2016). This drug was initially synthesized in China in 1986 and subsequently registered as Naphthoquine phosphate (NQP, Figure 1) in 1993. Like other 4-aminoquinolines, its schizontocidal activity likely results from inhibiting hemozoin biocrystallization in the parasite’s digestive vacuole during later stages. The combination of NQP and artemisinin, marketed under the brand name ARCO™^,^ represents an established antimalarial therapy that is available on the pharmaceutical market (Isba et al., 2015). Beyond its use in anti-malarial therapy, it has also been researched for potential applications in treating other diseases through drug repurposing. For instance, studies have shown that NQP exhibits growth inhibitory effects on *B. gibsoni in vitro* and on *B. rodhaini in vivo* (Ji et al., 2022). Our group discovered that it exhibited effective broad-spectrum antiviral activity against various coronaviruses in vitro, encompassing HCoV-229E, HCoV-OC43, and SARS-CoV-2 (Song et al., 2022). A filed patent disclosed that this compound might represent a promising candidate for broad-spectrum antimicrobial intervention (Zhang et al., 2016). However, its application in antimicrobial therapy, particularly against AB, remains largely unexplored. In this study, using high-throughput screening, we identified NQP to be a potential antibacterial candidate against AB. Further, we investigated the in vitro antibacterial activity as well as mechanisms of NQP in depth.

In this study, we utilized high-throughput screening to identify NQP as a promising antibacterial agent against AB. Additionally, we performed an extensive examination of NQP’s in vitro antibacterial activity specifically against AB, along with an exploration of the mechanisms responsible for its effectiveness.

## 2 Materials and methods

### 2.1 General reagents and microbial strains

Naphthoquine phosphate (NQP) was sourced from Shanghai New Hualian Pharmaceutical Co., Ltd. The 13 antibacterial agents included Meropenem trihydrate, Methicillin sodium salt, Vancomycin hydrochloride, Ceftazidime, Minocycline hydrochloride, Gentamicin (MCE, Shanghai, China); Cefepime (ACMEC, Shanghai, China); Imipenem (Aladdin, Shanghai, China); Erythromycin (Macklin, Shanghai, China), Levofloxacin (Solarbio, Beijing, China), Tigecycline, Polymyxin B, Polymyxin E (Yuanye, Shanghai, China). A custom compound library comprising 525 antimalarial compounds was obtained from MedChemExpress LLC.

The standard strains of *Staphylococcus aureus* (USA-300-R)*, Enterococcus faecalis* (HJP554)*, Acinetobacter baumannii* (LAC-4)*, Pseudomonas aeruginosa* (F291007)*, Klebsiella pneumoniae* (2146)*, Enterobacter cloacae* (ATCC BAA-2082) were preserved in our lab. The epidemiological cut-off value (ECOFF) for ceftazidime against *Acinetobacter baumannii* was set at 16 μg/mL with reference to the EUCAST 2025 data ECOFF. The MIC (The minimum inhibitory concentration) of the *Acinetobacter baumannii* LAC-4 to ceftazidime was 125 μg/mL, which is eight times the ECOFF value.

### 2.2 High throughput screening

To discover antimicrobial agents against ESKAPE pathogens, we screened a custom-made library of antimalarial compounds. Initially, log-phase ESKAPE bacteria were adjusted to 1 × 10^6^ CFU/mL in cation-adjusted Mueller-Hinton broth (Ca-MHB). A mixture of 100 μL bacterial suspension and 100 μL compound (~1 mg/mL per well) was plated in a 96-well format, incubated at 37°C for 16–18 h, and then assessed for bacterial precipitation. Polymyxin B, which is a widely recognized and clinically validated antibacterial agent for Gram-negative bacteria, was used as a positive control. Its minimum inhibitory concentration (MIC) against *Acinetobacter baumannii* was determined to be 0.98 μg/mL. In a second phase, the active compounds were further evaluated by microbroth dilution to determine their MICs, leading to the selection of NQP for advanced studies.

### 2.3 Determination of minimum inhibitor concentrations

Minimum inhibitor concentrations (MICs) were determined using the standard methods outlined by the Clinical and Laboratory Standards Institute (CLSI) (CLSI, 2018). Briefly, the bacteria were cultured to the logarithmic phase using Ca-MHB medium. NQP was prepared at a concentration of 2000 μg/mL in deionized water, filtered through a 0.22 μm membrane. Then serially twofold diluted with Ca-MHB medium to achieve concentrations ranging from 2000 to 0 μg/mL in a 96-well microtiter plate (100 μL per well). Bacterial suspensions (100 μL per well, with a final inoculum of 5 × 10^5^ CFU/mL) were added and incubated at 37°C for 24 h. Hundred μL of Ca-MHB medium was added to the wells for the 0 ug/mL control (solvent control). Concurrently, bacteria cultured solely in Ca-MHB medium (without NQP) served as the positive control. The MIC was defined as the lowest concentration of NQP that inhibited visible bacterial growth in Ca-MHB. All tests were repeated three times.

### 2.4 Cytotoxicity

The methods for cell culture and the CCK8 assay were performed as described previously (Cai et al., 2019; Wang et al., 2024). A549 cells were cultured in 90% Dulbecco’s Modified Eagle’s Medium (DMEM)-High Glucose Medium (Gibco), supplemented with 10% FBS (Ecosafene) and 1% penicillin (Gibco), and seeded into 96-well microtitre plates at a density of 3 × 10^4^ cells per well. Following overnight incubation at 37°C in a 5% CO_2_ atmosphere, the cells were washed twice with PBS. Approximately 200 μL of NQP diluted with DMEM series was added to each well. After a 24-h incubation at 37°C in a 5% CO_2_ atmosphere, the medium was aspirated and 110 μL of CCK8 solution was added to each well. After a further 4-h incubation, the absorbance was measured at 450 nm by an enzyme meter.

### 2.5 Hemolysis assay

The hemolysis assay was conducted as described previously (Yu et al., 2021). Whole blood was collected from Balb/c mice. 1.5 mL of murine whole blood was collected and centrifuged at 3000 rmp for 10 min at 4°C to separate erythrocytes from plasma. The erythrocytes were washed three times with 0.9% NaCl saline and then resuspended in saline. Resuspend the erythrocytes in saline to form a 2.5% v/v suspension, then add 700 μL of this suspension to a 1.5 mL centrifuge tube, centrifuge at 3000 rpm for 10 min and discard the supernatant. The test drug was dissolved in saline and 700 μL of varying concentrations of NQP was added to the erythrocyte sediment. An equal volume of saline was added to the negative control (C–), and an equal volume of distilled water was added to the positive control (C+) to induce osmotic hemolysis. Incubate at 37°C for 1 h and centrifuge again to pellet the intact erythrocytes. Measure the absorbance of the supernatant at 540 nm using an enzyme meter. Hemolysis is expressed as the percentage of erythrocytes and is calculated using the formula: Hemolysis (%) = (A-A_C-_)/ (A_C+_-A_C-_) × 100%. A is the absorbance of the test sample, A_C-_ is the absorbance of the negative control and A_C+_ is the absorbance of the positive control. The experiment was repeated three times for statistical analysis.

### 2.6 Time-kill and growth curve assay

The Time-kill assay was conducted as described in the literature (Wang et al., 2023). The bacterial inoculum (approximately 5 × 10^5^ CFU/mL) was introduced into the control and NQP-treated groups (1/2MIC, MIC, 2MIC, 4MIC, 8MIC) to a final volume of 10 mL. These cultures were then incubated at 37°C in an incubator. At the specified time intervals (0, 0.5, 1, 2, 4, 6, 8, 23, and 24 h), 100 μL samples were collected from each group and subjected to a 10-fold serial dilution. Subsequently, 100 μL of each dilution was spread onto Mueller-Hinton Agar (MHA) plates and incubated for 24 h at 37°C.

To further assess NQP’s growth curves, 200 μL sample of each group was collected and transferred to a 96-well plate within the above time points. The absorbance value at 600 nm was then measured using an enzyme meter. Growth curves were generated based on the recorded absorbance values. Referring to this literature (Wang et al., 2010), the bacterial growth inhibition percentage (%) was calculated using the formula: Inhibition (%) = [(Ac-At)/Ac]x100%. Ac represents the average of three absorbance values at 600 nm for the control group, and At represents the absorbance value at 600 nm for the samples. The IC_50_ value was calculated using the linear relation between the inhibitory probability and concentration logarithm.

### 2.7 Infection and antibacterial activity in A549

Reference to previous studies (Genteluci et al., 2020), A549 cells were inoculated into 96-well plates (2 × 10^4^ cells/well) and incubated for 24 h at 37°C in a 5% CO_2_ incubator. AB LAC-4 suspension was added to the cells at a multiplicity of infection (MOI) = 200 and after 2 h of LAC-4 infection at 37°C, A549 cells were washed three times with PBS to remove the free bacterial cells, and then treated with NQP of 1/2MIC, MIC, 2MIC, 4MIC, 8MIC for an additional 2 h at 37°C. Cells were washed three times with PBS and lysed in 100 μL cold PBS containing 0.1% Triton X-100. Counts of surviving bacteria were determined by inoculating serial dilutions of cell lysates on MHA agar.

### 2.8 Membrane permeability

The integrity of cell membranes was assessed using propidium iodide (PI) (Deng et al., 2020). AB LAC-4 was treated with NQP, and the cultures were incubated at 37°C. The fluorescence intensity of PI was observed, photographed using a fluorescence microscope and quantified using ImageJ software.

An equivalent number of cells were collected, incubated with NQP at 37°C for 1 h, and then centrifuged to obtain the supernatant. The ATP concentration in the supernatant was determined using an ATP assay kit (Beyotime, A22066) (Spari and Beldi, 2020). Briefly, luciferase catalyzed the reaction between ATP and D-luciferin, producing light that was measured using a multifunctional plate reader (Spectra Max i3x; Molecular Devices, Shanghai, China).

The bacterial suspension was adjusted to an OD_600_ of 1.0 with medium, and ortho-nitrophenyl-*β*-D-galactopyranoside (ONPG) was added, followed by the addition of a sterile aqueous solution of NQP adjusted to 1/2 MIC and MIC concentrations. Sterile water was added to the negative control group. The samples were incubated for 1 h at 37°C with a final concentration of 3 mM ONPG. Following the addition of the samples to a 96-well plate, protected from light, the absorbance of the sample solutions was measured at 420 nm (Eumkeb and Chukrathok, 2013).

### 2.9 Fluorimeter assay for membrane potential

The experimental method employed herein was modified based on previous descriptions (Buttress et al., 2022). The bacterial suspension was adjusted to an OD_600_ of 1.0. Disc3(5) was added and the mixture was incubated in a biochemical incubator at 37°C, shielded from light for 1.5 h. Subsequently, different concentrations of NQP (1/2 MIC and MIC) were added to the positive control group, while sterile water was added to the negative control group. Incubate for an additional 15 min at 37°C. The final concentration of Disc3(5) was 50 nM. Samples were transferred to 96-well plates, protected from light. In the fluorescence plate reader, the excitation wavelength was adjusted to 622 nm, while the emission wavelength was set to 670 nm, in order to accurately measure any alterations in the fluorescence intensity of the samples.

### 2.10 Scanning electron and transmission electron microscopy

Scanning electron microscopy (SEM) and transmission electron microscopy (TEM) were utilized to assess the effects of NQP on AB LAC-4. Bacteria were cultured and treated with the MIC for 1 h in Ca-MHB, following the same procedure as in the time-kill studies.

For the SEM analysis, samples were immediately fixed in a 2 mL centrifuge tube containing 2.5% glutaraldehyde and stored at 4°C for 12–24 h. The fixative was aspirated, and the samples were rinsed three times with PBS buffer (pH 7.0), followed by dehydration through a series of increasing ethanol concentrations. The dehydrated samples were placed in a critical point dryer to dry, then affixed to the sample stage with conductive carbon glue, t and coated with platinum using an ion-sputtering device for 120 s. Finally, all samples were visualized using a SEM (Hitachi Regulus 8,100, Japan).

For TEM analysis, following the initial fixation, the fixative was aspirated and the samples were rinsed three times with PBS buffer (pH 7.4). The samples were post-fixed with 1% OsO_4_ solution for 1–2 h. After removing the OsO_4_, the samples are rinsed three times with 0.1 M PBS (pH 7.4). Subsequently, the samples underwent dehydration, resin infiltration and embedding, polymerization, ultra-thin sectioning, and staining. Finally, the samples were visualized using a TEM (Hitachi HT7800, Japan).

### 2.11 Evaluation of oxidative stress within bacteria

#### 2.11.1 Determination of reactive oxygen species (ROS) by luciferase assay

The bacterial suspension was adjusted to OD_600_ = 1.0, NQP was added and incubated at 37°C for 1 h. The concentration of NQP was adjusted to 0, 1/2MIC, MIC, 2MIC, 4MIC and 8MIC. Bacterial precipitate was collected by centrifugation, and the precipitate was resuspended in DCFH-DA (10 μM) diluted in the culture medium. Incubation was continued in the incubator at 37°C for 20 min to allow the probe to enter the cells. Post-reaction, the cells were washed twice with 0.05% Poloxamer-containing saline to thoroughly remove any probe that had not entered the cells. The samples were transferred to a 96-well plate at 200 μL per well, and the fluorescence intensity (excitation wavelength 488 nm; emission wavelength 525 nm) was measured by a multifunctional enzyme labeling instrument (SpectraMax i3x, Shanghai) (Zheng et al., 2019).

#### 2.11.2 Measurement of NADH levels

Sample collection was conducted according to section 2.11.1. The specified cells were collected and intracellular NADH levels were quantified by using an NAD+/NADH assay kit (Beyotime, S0175) with WST-8, a superior substitute for MTT, following the manufacturer’s protocol (Ma et al., 2022). NADH levels were determined by incubating cell lysates at 60°C for 30 min to eliminate NAD+, followed by the conversion of WST-8 to formazan in the presence of NADH and 1-mPMS (1-Methoxy-5-methylphenyldiazonium Methyl Sulfate). The total NADH content was assessed by measuring absorbance at 450 nm using a multifunctional enzyme labeling instrument (SpectraMax i3x; Molecular Devices, Shanghai, China).

#### 2.11.3 Quantification of H_2_O_2_ production

Sample collection was performed according to the procedure outlined in section 2.11.1. Briefly, the organisms were added to 400 μL of lysis buffer provided in the H_2_O_2_ Assay Kit and lysed on ice for 1 h. After the organisms had been lysed, the supernatant was collected and intracellular H_2_O_2_ levels were measured using a Hydrogen Peroxide Assay Kit (Beyotime Institute of Biotechnology, Shanghai, China, S0038) (Zhou et al., 2022).

#### 2.11.4 Evaluation of superoxide dismutase (SOD) and malondialdehyde (MDA) levels

A Total Superoxide Dismutase (T-SOD) Assay kit with WST-8 (cat. S0101; Beyotime Institute of Biotechnology) was utilized to determine the intracellular SOD level following the manufacturer’s protocols. A Lipid Peroxidation MDA Assay kit (cat KTB9050; Abbkine) was employed to measure MDA levels (Merghni et al., 2023).

### 2.12 Transcriptomics

AB LAC-4 was cultured to logarithmic growth stage, and the bacterial concentration was adjusted to OD_600_ ≈ 1.0 using Ca-MHB medium. Subsequently, the culture was treated with NQP at the MIC for 1 h, with an untreated control serving as the negative control. Finally, the bacterial cells were centrifuged, and the pellet was collected for RNA sequencing.

Differential expression analysis of the digital gene expression data was conducted using the DESeq2 R package (version 1.20.0), employing a negative binomial distribution for modeling and determination (Love et al., 2014). Genes with adjusted *p* values < 0.05, as determined using the Benjamini-Hochberg procedure, were considered significantly differentially expressed. GO and KEGG enrichment analyses were performed using GOseq R software and KOBAS software to identify the biological functions and pathways predominantly impacted by the DEGs (Mao et al., 2005; Young et al., 2010).

### 2.13 Validation of RNA sequencing

RNA-seq samples were prepared, and RT-qPCR analysis was conducted on eight randomly selected differentially expressed genes using the One Step PrimeScript RT-PCR Kit (Perfect Real Time) to validate the RNA-seq results. These genes, which were located in different KEGG pathways and had a fold change greater than 1.5, included 4 up-regulated and 4 down-regulated genes. RT-qPCR was carried out on a CFX opus 96 system (Bio-Rad, Singapore) with the following thermal profile: one cycle of reverse transcription at 42°C for 5 min and 95°C for 10 s, followed by 39 cycles of denaturation at 95°C for 5 s and annealing at 55°C for 20 s. A total of 39 cycles were conducted, with each PCR reaction repeated three times. Gene expression levels was calculated using the 2^-ΔΔCT^ method with 16 s rRNA as the endogenous reference.

### 2.14 Statistical analysis

Statistical analysis was performed using IBM SPSS Statistics 26, and graphs were generated using GraphPad Prism version 8. Data are presented as mean ± standard deviation (SD). For data following a normal distribution, one-way analysis of variance (ANOVA) was used to analyze the differences between the means of the control and treatment groups. For data that did not follow a normal distribution, the non-parametric Kruskal-Wallis one-way analysis was employed. A *p*-value less than 0.05 (*p* < 0.05) was considered statistically significant.

## 3 Results

### 3.1 Antibiotic susceptibility

We conducted a high-throughput screening of an antimalarial compound library utilizing AB LAC-4 in order to identify promising antimicrobial molecules. After analyzing two rounds of screening data, we selected NQP for further study. To further assess the bacteriostatic activity of NQP, we evaluated its efficacy against several ESKAPE pathogens. As listed in Table 1, NQP demonstrated potent bactericidal activity against AB, with MIC of 62.5 μg/mL. Notably, NQP also exhibited comparable bactericidal activity against Gram- positive bacteria of *Staphylococcus aureus* and *Enterococcus faecalis*, with MICs of 31.25 μg/mL and 31.25 μg/mL, respectively. Furthermore, Table 1 displays the MIC values of some clinically significant antibiotics against these bacterial strains. Ceftazidime exhibited low antimicrobial activity against *Acinetobacter baumannii* strain LAC-4, with a MIC of 125 μg/mL.

**Table 1:** Minimum inhibitory concentrations of NQP and antibiotics on ESKAPE (μg/mL).

Compounds	Gram-positive bacteria		Gram-negative bacteria			
	*Staphylococcus aureus (USA-300-R)*	*Enterococcus faecalis (HJP554)*	*Acinetobacter baumannii (LAC-4)*	*Pseudomonas aeruginosa (F291007)*	*Klebsiella pneumoniae (2146)*	*Enterobacter cloacae (ATCC BAA-2082)*
NQP	31.25	31.25	62.5	>1,000	>1,000	500
Polymyxin B	125	1,000	0.98	3.9	1.9	3.9
Polymyxin E	250	>1,000	0.98	3.9	1.9	1.95
Meropenem	15.63	>500	3.9	250	7.8	125
Tigecycline	0.98	0.06	3.9	15.63	7.8	>1,000
Cefepime	250	>1,000	7.81	7.81	125	>1,000
Imipenem	15.63	>1,000	1.95	1,000	15.63	>1,000
Methicillin	62.5	>1,000	500	>1,000	>1,000	>1,000
Vancomycin hydrochloride	3.9	>1,000	125	>1,000	1,000	>1,000
Erythromycin	250	>250	3.9	250	>250	>250
Gentamycin	7.81	125	125	>1,000	>1,000	3.9
Levofloxacin	15.63	62.5	1.95	62.5	250	>1,000
Minocycline	0.5	0.13	0.03	62.5	250	>1,000
Ceftazidime	250	>1,000	125	31.25	500	>1,000

### 3.2 Cytotoxicity

**Figure 2:**
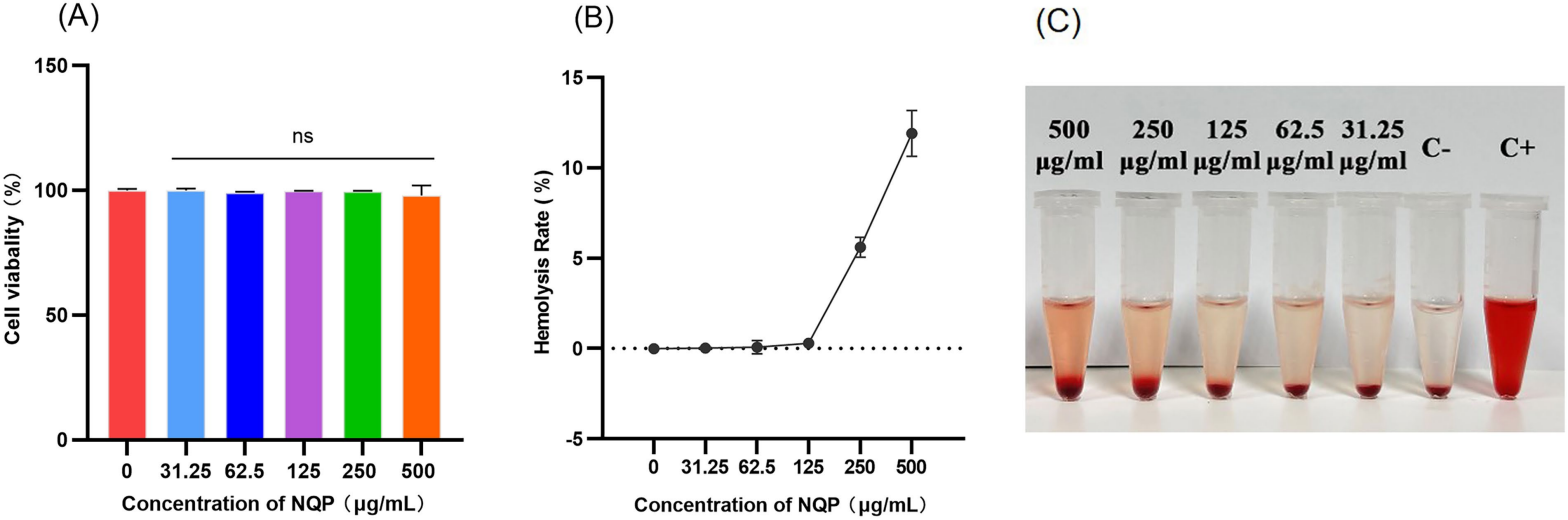
**(A)** Cytotoxic effect of different concentrations of NQP on A549 cells. Ns: no significance (*p* > 0.05), compared to the control group. **(B)** The hemolysis assay: the hemolysis percentage of NQP at different concentrations. **(C)** Visualization of tubes after incubation and centrifugation. C–: the negative control; C+: the positive control.

The cytotoxicity of drugs serves as a pivotal factor that may restrict their therapeutic utilization. The cytotoxic effects of NQP on A549 cells were assessed using the CCK8 assay. NQP exhibited no cytotoxicity toward A549 cells, even at a concentration of 500 μg/mL, which is 8 times greater than the MIC against the bacteria (Figure 2A). Furthermore, we evaluated the hemolytic activity of NQP toward erythrocytes (Figures 2B,C). No significant hemolysis was observed in erythrocytes at a concentration of 125 μg/mL NQP, which was significantly below the ASTM F756-17 safety threshold (<2%) and exceeded the MIC for the AB LAC-4 strain (ASTM, 2017). These findings indicate that NQP can be used within a therapeutic concentration range without causing undue toxicity.

### 3.3 MIC and antibacterial kinetics

**Figure 3:**
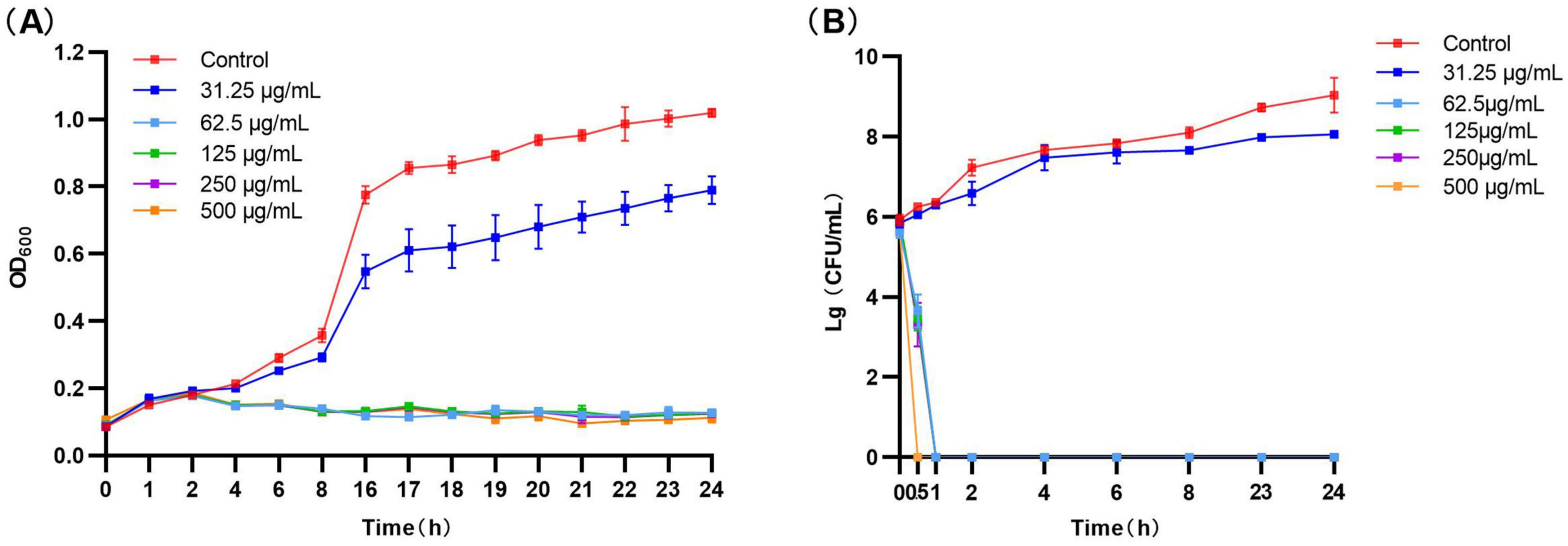
**(A)** Bacterial growth kinetics of NQP against *Acinetobacter baumannii*. **(B)** Time-kill test results curve for NQP against *Acinetobacter baumannii* LAC-4.

As show in Figure 3, NQP showed good *in vitro* antimicrobial activity against AB, with MIC of 62.5 μg/mL and an IC_50_ of 38.56 μg/mL.

The inhibition kinetics in Figure 3 revealed that the growth trend of AB was comparable to that of the control group when exposed to 31.25 μg/mL NQP. At 62.5 μg/mL, there was a sharp decrease in bacterial count, with a complete bactericidal effect observed within 1 h. At concentrations of NQP ≥ 125 μg/mL, all bacterial cells were killed within 0.5 h.

### 3.4 Antibacterial activity on infected cells

To determine the effect of NQP on intracellular bacteria, we conducted subsequent experiments using AB LAC-4. The A549 cell line, which is derived from human alveolar epithelial cells, was employed to establish the infection model.

**Figure 4:**
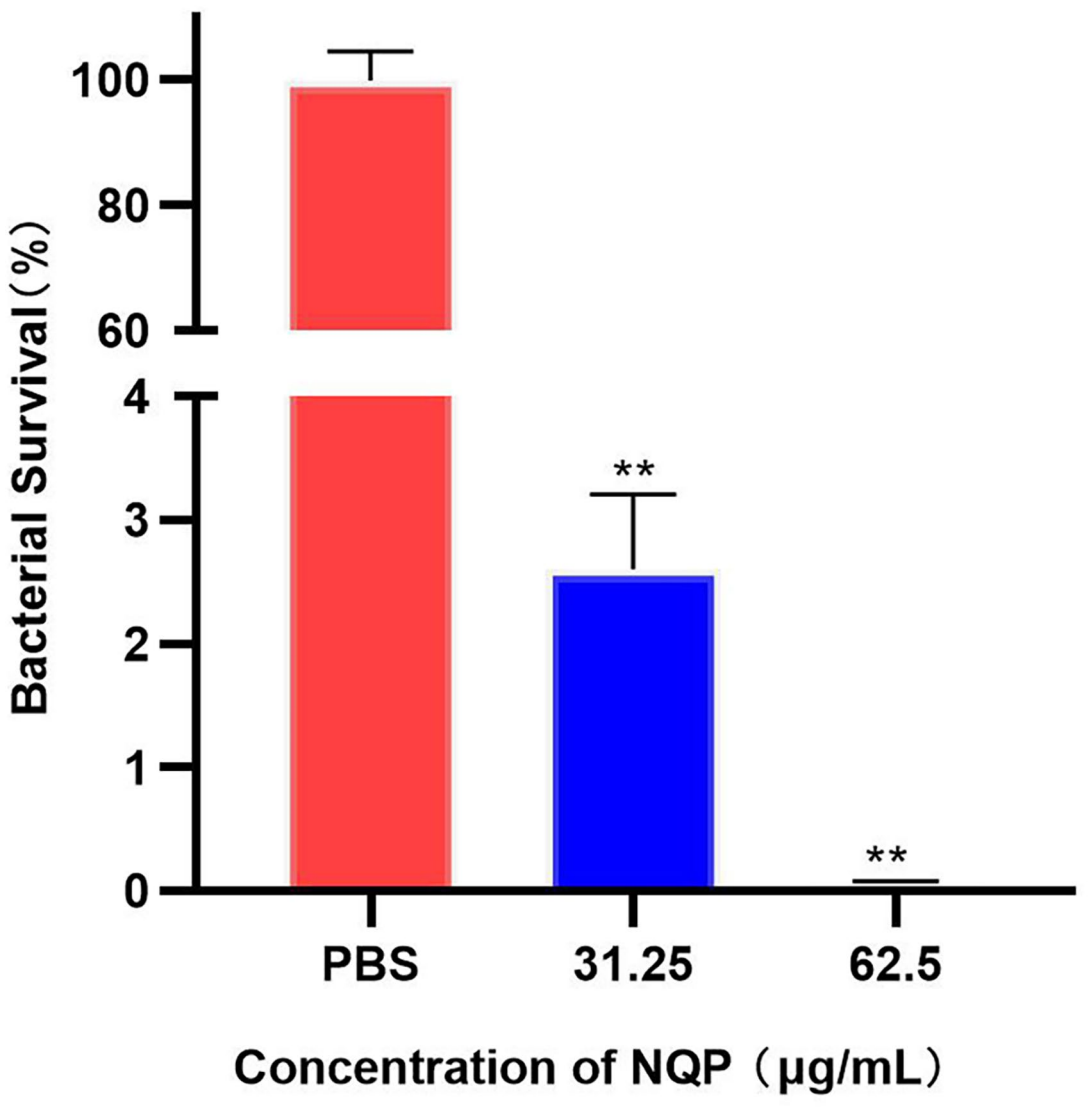
Bacterial survival fraction of *Acinetobacter baumannii* LAC-4 treated with different concentrations of NQP. PBS served as the negative control. Level of significance (*p* < 0.01) is indicated by **.

Based on this, antibacterial activity experiments were conducted, which revealed that approximately 29,833 ± 1,365 bacteria adhered to the surface of A549 cells 2 h post-infection. Following treatment with 31.25 μg/mL NQP, both the number of LAC-4 cells adhering to and surviving on the A549 cells were significantly reduced compared to the control group (*p* < 0.0001). After treatment with 62.5 μg/mL NQP, only 0.06% survival was observed (Figure 4).

### 3.5 NQP function on membrane damage

**Figure 5:**
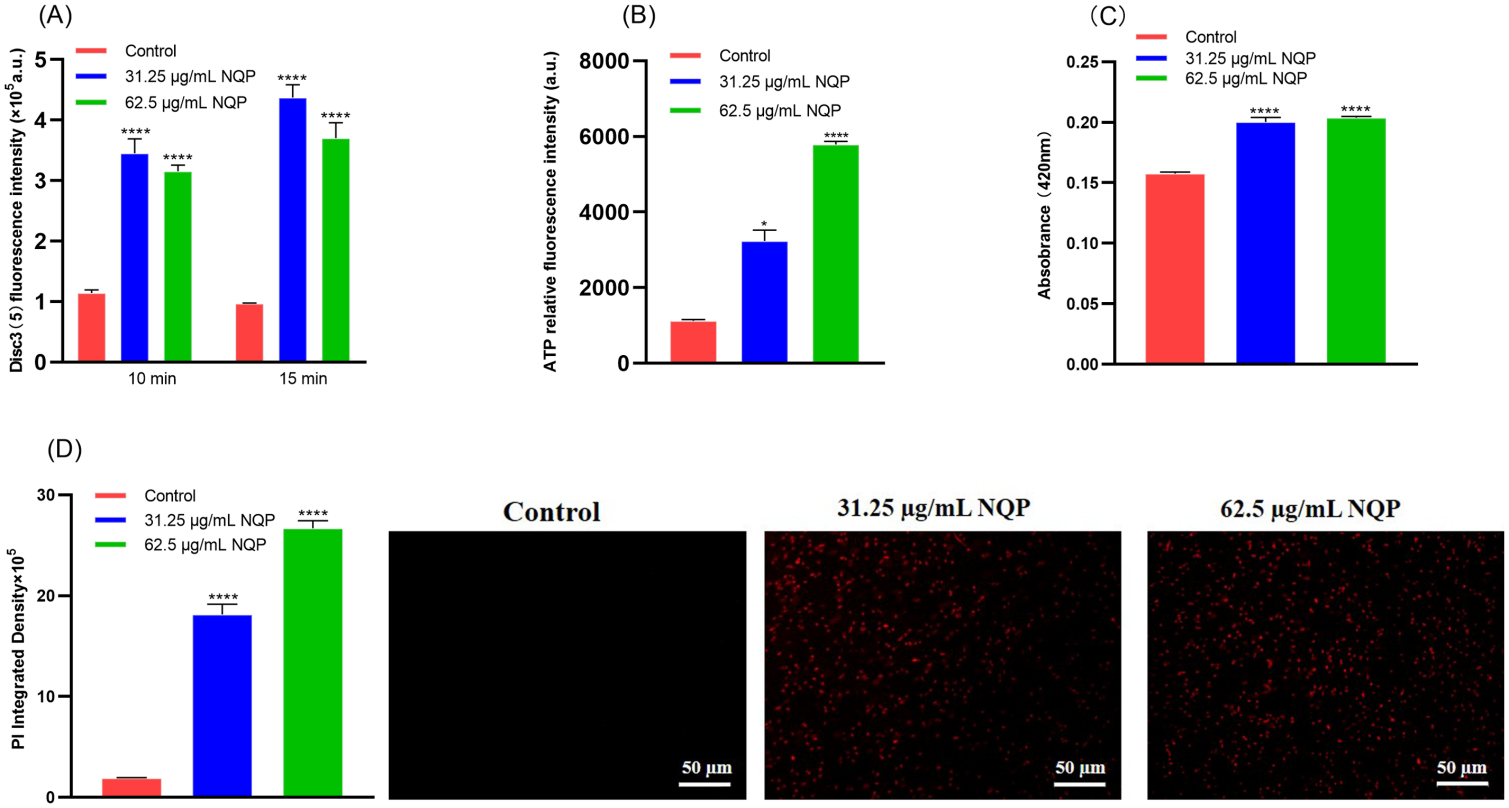
Detection of NQP-induced damage to the cell membranes of *Acinetobacter baumannii* LAC-4. **(A)** Cell membrane potential of *Acinetobacter baumannii* LAC-4 cells monitored with DiSC3(5) after incubation with NQP. **(B)** Intracellular ATP in bacterial cells treated with different concentrations of NQP. **(C)** The absorbsance at 420 nm in ONPG hydrolysis test. **(D)** The fluorescence intensity of PI was photographed using a fluorescence microscope and quantified using ImageJ software. Error bars show the SDs of experiments performed in triplicate. * Significantly different (*p* < 0.05); **** Significantly different (*p* < 0.0001).

We employed the fluorescent dye Disc3(5), known for its sensitivity to alterations in membrane potential, to detect the depolarizing effect. The results are presented in Figure 5A. The fluorescence intensity observed in the NQP-treated group was significantly higher than that in the control group, indicative of membrane depolarization. Moreover, the degree of membrane potential depolarization increased with higher concentrations of NQP.

We further conducted assays for ONPG excretion, ATP release, and PI staining assays. As illustrated in Figure 5B, NQP increased extracellular ATP release in a dose-dependent manner. In Figure 5C, the absorbance measured at 420 nm, which serves as an indicator of *β*-galactosidase activity in the culture medium, was significantly elevated in the NQP-treated group compared to the control group. This increase was proportional to the higher concentrations of NQP used. Concurrently, when AB was treated with the 1/2MIC and MIC of NQP and stained with propidium iodide (PI), an increase in the number of dead (red) bacteria was observed (Figure 5D). These results demonstrated that the permeability of AB cell membranes increased following NQP treatment.

### 3.6 Morphological changes of AB LAC-4

**Figure 6:**
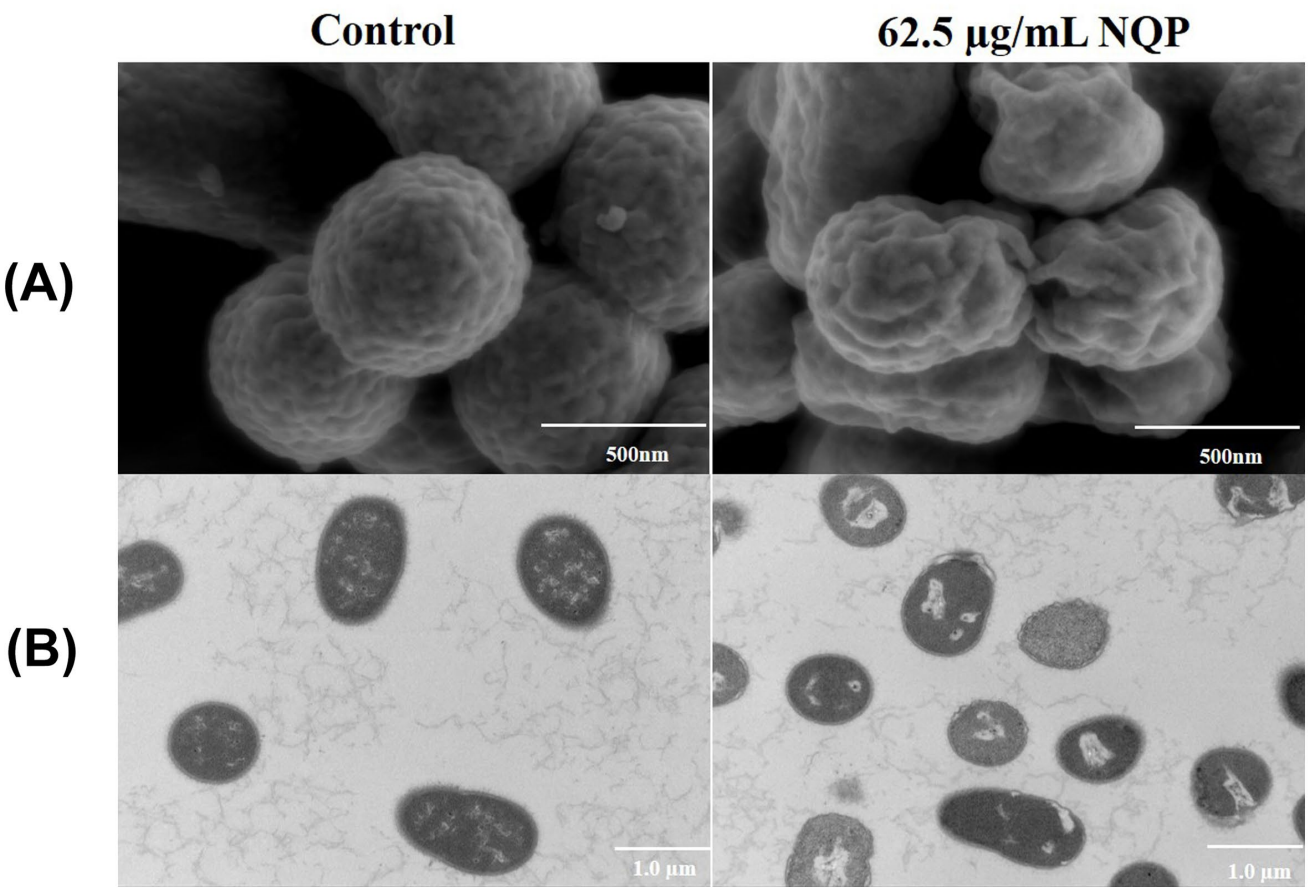
**(A)** Scanning electron microscopy (SEM) and **(B)** transmission electron microscopy (TEM) images of *Acinetobacter baumannii* LAC-4 treated with 62.5 μg/mL NQP. The control group shows bacteria untreated with NQP.

SEM images revealed that the bacteria in the untreated control group, exhibit pristine morphology, characterized by regular shapes, consistent sizes, and clearly discernible surface features. In contrast, SEM images of the NQP-treated group displayed bacteria exhibiting wrinkled and diversely deformed surfaces (Figure 6A), offering compelling evidence of NQP’s ability to disrupt bacterial membrane integrity.

TEM images revealed that the bacteria in the control group possessed regular morphology, with smooth and well-defined edges, and a consistent distribution of intracellular contents. While NQP-treated bacteria exhibited vacuolation, plasma membrane detachment, irregular edges, and deformation of the cell membrane (Figure 6B).

### 3.7 ROS-mediated impairment of activity

**Figure 7:**
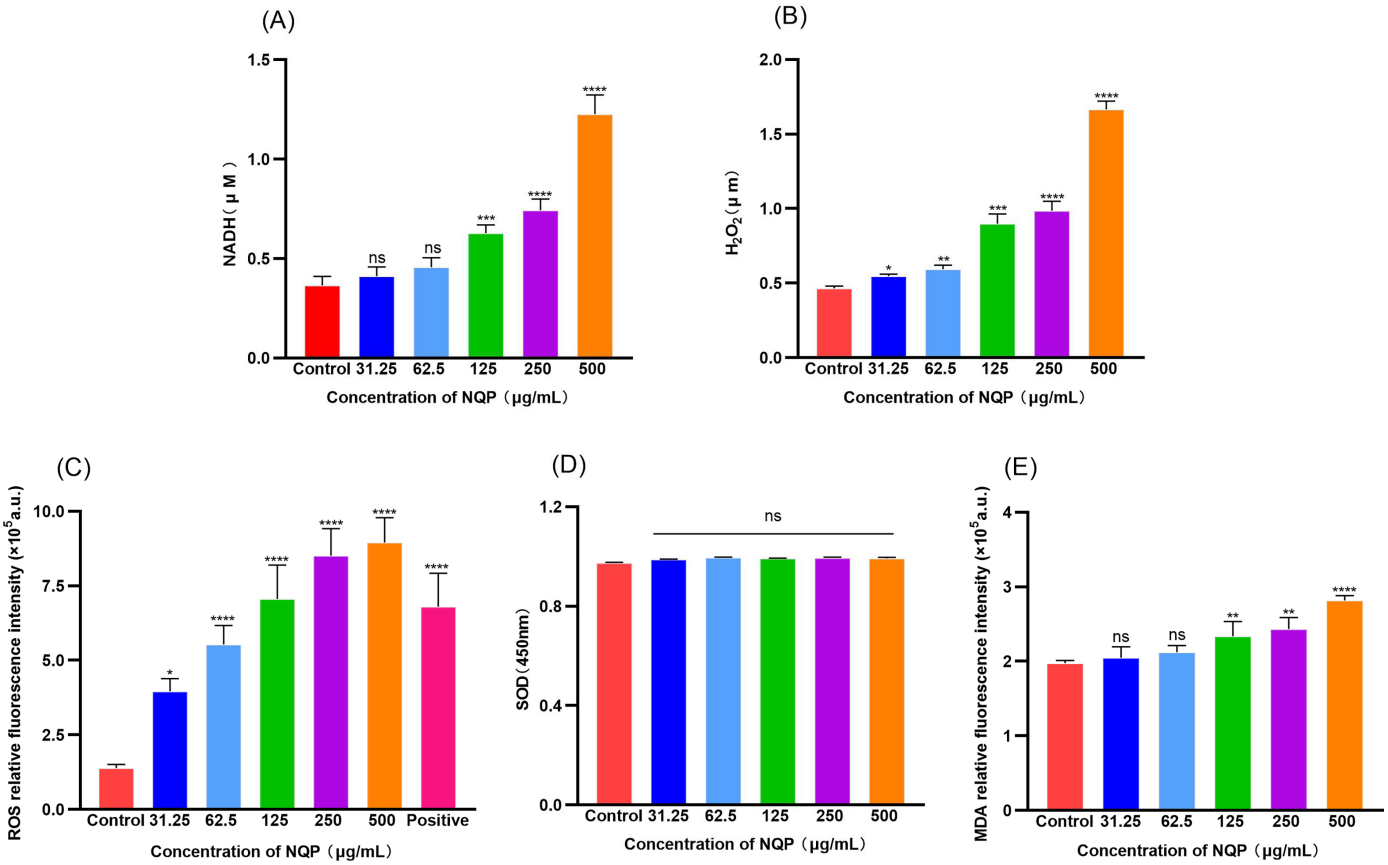
Detection of NQP-induced oxidative damage mediated by reactive oxygen species in *Acinetobacter baumannii* LAC-4. **(A)** NADH; **(B)** H_2_O_2_; **(C)** ROS; **(D)** SOD; **(E)** MDA. Error bars show the SDs of experiments performed in triplicate. ns: no significance (*P* > 0.05); * Significantly different (*p* < 0.05); ** Significantly different (*p* < 0.01); *** Significantly different (*p* < 0.001); **** Significantly different (*p* < 0.0001).

Figure 7 illustrates that NADH levels in the NQP-treated group (Figure 7A) were higher than that in the control group. As a product of TCA cycle, the increased levels of NADH stimulate the process of oxidative phosphorylation. Furthermore, a substantial increase in hydrogen peroxide levels was noted in the assays (Figure 7B), accompanied by a marked elevation in superoxide ROS levels (Figure 7C).

Excessive ROS will cause damage to intracellular DNA, iron–sulfur protein clusters, lipids, and other vital cellular components within bacterial cells (Vaishampayan and Grohmann, 2021). The level of SOD secreted by the bacteria did not increase significantly (Figure 7D), and was therefore insufficient to counteract the damage inflicted by excessive ROS. Consequently, NQP significantly elevated the intracellular ROS levels in the bacteria, hastening bacterial death. The impact of NQP on lipid peroxidation in AB is depicted in Figure 7E. The malondialdehyde (MDA) content in AB progressively increased, as ROS accumulation induced lipid peroxidation within the cell membrane. This, in turn, compromised the membrane’s integrity and enhanced its permeability.

### 3.8 Differently expressed genes

**Figure 8:**
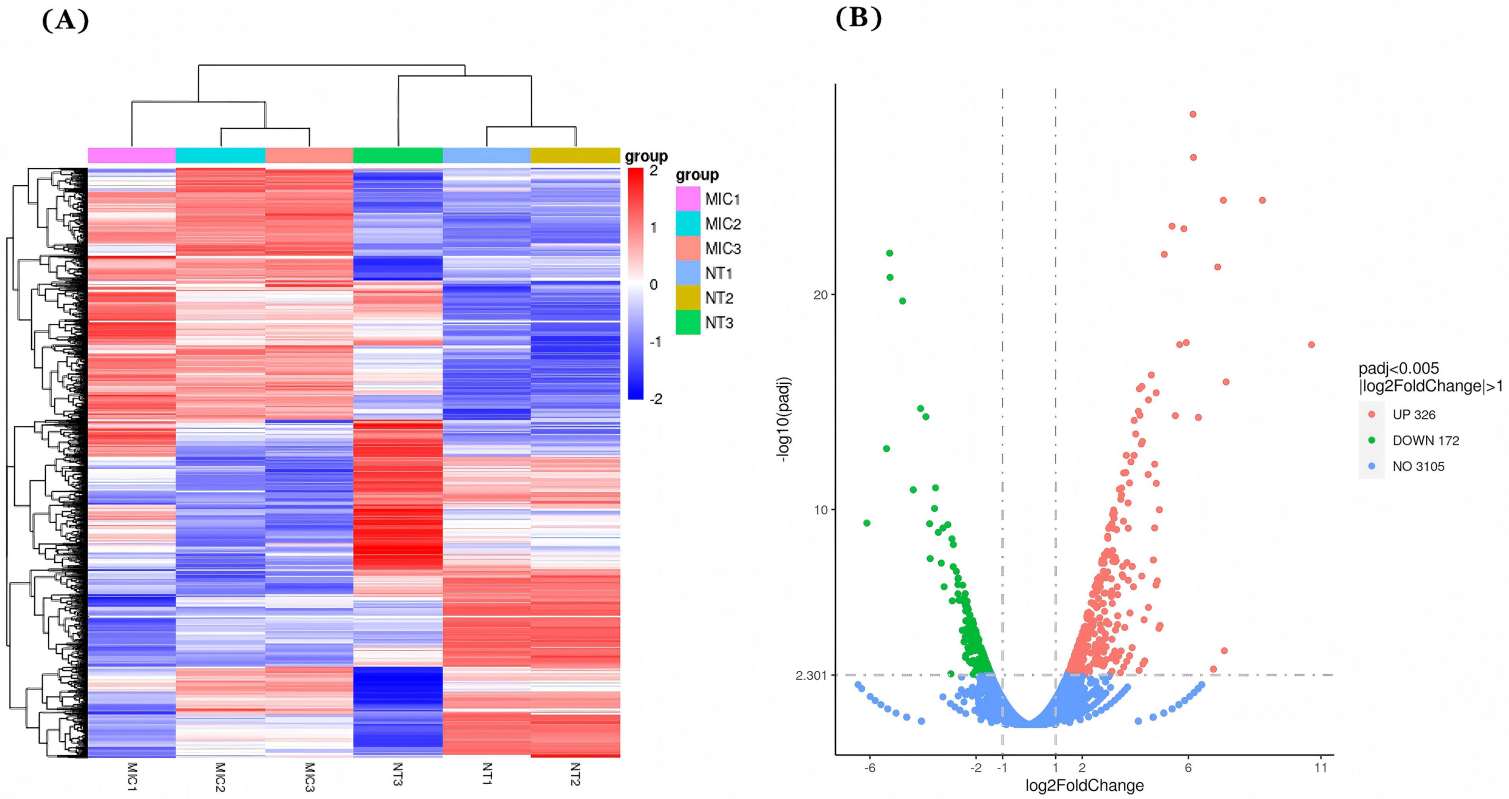
Differentially expressed genes (DEGs) after NQP treatment of *Acinetobacter baumannii* LAC-4. **(A)** Expression heatmap of 498 DEGs analyzed by hierarchical clustering, where red indicates relatively high expression levels and blue indicates relatively low expression levels. **(B)** Volcano map of DEGs with 326 up-regulated genes (red dots) and 172 down-regulated genes (green dots). Blue dots indicate genes with no significant change in expression. MIC: Treated with a concentration of 62.5 μg/mL NQP; NT: The Control group.

After conducting the differential expression analysis, 498 differentially expressed genes (DEGs) [*P*-adj ≤ 0.05 | log_2_Fold Change| ≥ l.0] were identified. Among these, 326 genes were up-regulated and 172 genes were down-regulated in the administered group (MIC) compared to the blank group (NT) (Figure 8).

### 3.9 RNA sequencing validation

**Figure 9:**
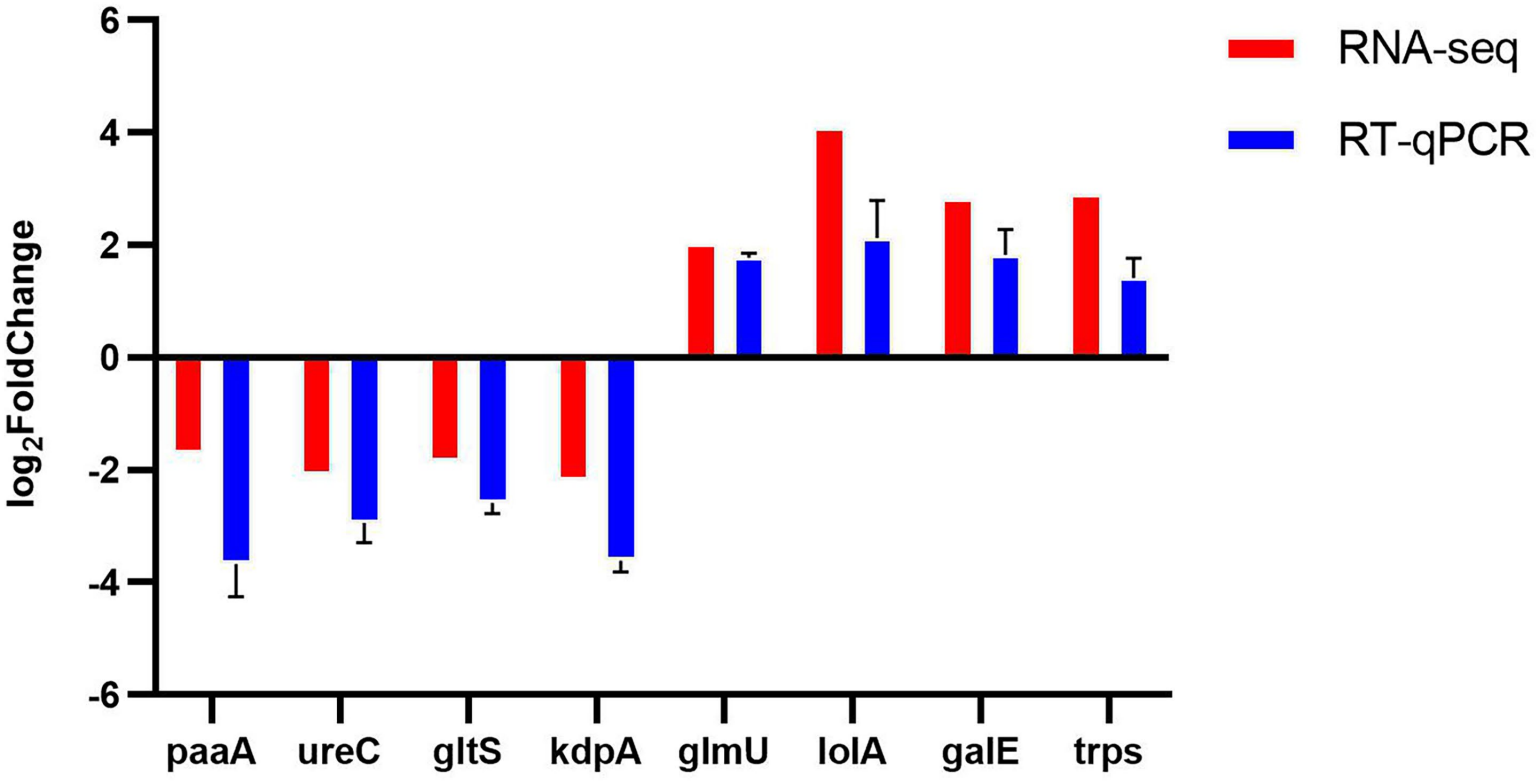
Eight differentially expressed genes (DEGs) after NQP treatment of *Acinetobacter baumannii* LAC-4 identified by RNA-seq were validated using RT-qPCR. Red: RNA-seq; Blue: RT-qPCR. Error bars show the SDs of experiments performed in triplicate. 16s rRNA was used as the reference gene for normalization.

Eight genes, comprising four up-regulated and four down-regulated according to RNA sequencing and transcriptomic analyses, were subjected to reverse transcription-quantitative polymerase chain reaction (RT-qPCR) validation (Figure 9). This RT-qPCR analysis aimed to confirm the findings from the RNA sequencing and transcriptomic studies. The expression trends of these genes, as observed in RT-qPCR and RNA sequencing, were consistent, thereby affirming the reliability of the RNA sequencing data. Discrepancies between RNA-seq and RT-qPCR results may be attributed to factors such as the small sample size, RNA degradation, and low levels of gene expression.

### 3.10 Effect of NQP on gene expression in AB LAC-4

**Figure 10:**
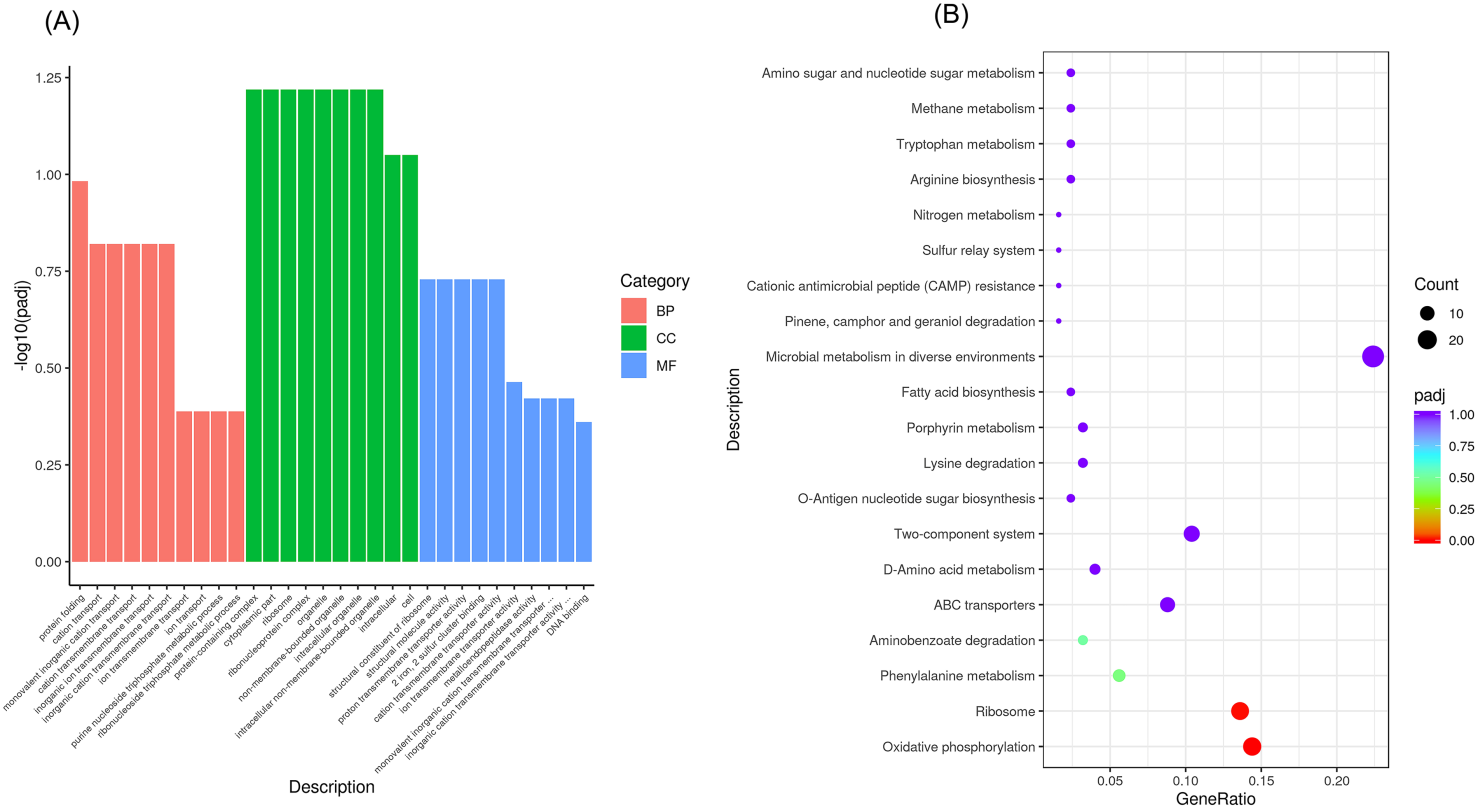
GO functional enrichment analysis and KEGG pathway enrichment after NQP treatment of *Acinetobacter baumannii* LAC-4. For genes with [|log_2_FC| > 1 and *p* < 0.05], GO and KEGG enrichment analyses were performed. **(A)** Bar plots display the top 10 significantly enriched terms. **(B)** Bubble plots show the top 20 pathways, where bubble size represents gene count and color indicates *p*-value. BP: Biological Process; CC: Cellular Component; MF: Molecular Function.

GO analysis revealed alterations in biological processes, molecular functions, and cellular composition, indicating that upregulated DEGS are predominantly implicated in molecular functions and the metabolism of cytoplasmic and organellar components as well as genetic material. The results demonstrated 188 annotations related to biological process functions, 21 annotations related to cellular composition and 112 annotation related to molecular functions (Figure 10A). KEGG pathway analysis indicated that the pathways enriched by differentially expressed genes were predominantly Oxidative phosphorylation, ribosomes, ABC transporters, as well as and metabolic pathways, including those for tyrosine, phenylalanine, and pyruvate metabolism (Figure 10B).

## 4 Discussion

In recent years, the threat of antimicrobial drug resistance has progressively narrowed the options available for treating infectious diseases. The definitive resolution to this pressing matter requires the development and introduction of new classes of compounds tailored to combat these resilient drug-resistant bacteria. The potential for drug repurposing or combinations appears as a promising approach to tackle the development of resistance in bacterial infectious diseases, as evidenced in studies.

### 4.1 *In vitro* antibacterial activity

To further evaluate the antimicrobial activity of NQP, we determined the MICs of NQP against additional multidrug-resistant strains. AB exhibits resistance to clinically standard antibiotics, including methicillin, vancomycin, ceftazidime, and gentamicin. LAC-4, a well-characterized, extensively drug-resistant strain of AB, serves as an invaluable model for modeling real-world therapeutic challenges in AB infections. In contrast, NQP demonstrated potent bacteriostatic effects against Gram-negative multidrug-resistant AB isolates. Additionally, we assessed the antibacterial activity of common clinical antibiotics against ESKAPE pathogens and preliminarily evaluated the broad-spectrum potential of NQP to inform subsequent research directions.

Naphthoquine phosphate (NQP), a novel antimalarial drug exhibiting favorable efficacy, has been consistently utilized in the clinical management of malaria (Moore et al., 2016). It belongs to the quinoline class of antimalarial compounds, a category that encompasses various other drugs such as chloroquine (CQ), hydroxychloroquine (HCQ), and amodiquine, among others (Deshp and Kuppast, 2016). It has been reported that chloroquine exhibit some antibacterial activity, albeit only at high concentrations, due to their structural resemblance to quinolone antibacterials (Nguyen et al., 2006; Rolain et al., 2007). Literature has demonstrated that Amodiaquine, an antimalarial drug that shares the closest structural similarity with NQP, provides protection against anthrax through a host-directed antimicrobial mechanism (Martchenko Shilman et al., 2021). However, it lacks antimicrobial activity against other bacterial strains (Kamurai et al., 2020). Additionally, there have been no reported instances of 4-aminoquinoline analogs exhibiting activity against AB. In our experiment, NQP demonstrated a notable inhibitory effect on the growth of AB. The antimicrobial kinetic curve indicated that treatment with NQP at a concentration of 62.5 μg/mL completely killed AB within 1 h. Drawing on comprehensive experimental data and published literature, NQP demonstrates superior antibacterial activity to other antimalarial drugs.

### 4.2 Intracellular bacteriostatic activity

Although it is already a marketed drug and its safety is well-established, we conducted cellular-level tests for toxicity and hemolysis to gain a deeper understanding of its antimicrobial mechanism. At a concentration of 125 μg/mL, which surpasses the minimum inhibitory concentration (MIC), NQP exhibited no toxicity to A549 cells and did not induce hemolysis in erythrocytes. This suggests that the cytotoxic side-effects of NQP on eukaryotic cells remain within an acceptable range for therapeutic efficacy. Additionally, we treated A549 cells infected with the LAC-4 strain with NQP and observed a significant reduction in the survival rate of bacteria in the treated group compared to the control group. Treatment with 62.5 μg/mL naphthoquin phosphate (NQP) resulted in near-complete eradication of bacterial populations, corroborating the findings from the time-kill kinetics analysis. This suggests that NQP may possess the capability to disrupt intracellular colonization during bacterial infections.

### 4.3 Damage to cell membranes

To elucidate its antimicrobial mechanism, we conducted a series of experiments to investigate potential pathways and modes of action. The bacterial cell membrane hosts numerous enzyme systems that are crucial for physiological activities. Therefore, damage to the bacterial membrane can directly or indirectly impair the functioning of these metabolic processes, ultimately leading to bacterial death (Chen et al., 2024). Antimicrobial drugs can affect the function of bacteria by disrupting the structure of bacterial cell membrane and impairing its selective permeability function (Abushaheen et al., 2020). In general, damage to the bacterial cell membrane leads to alterations in membrane potential. Disc3(5), a membrane potential sensitive probe, accumulates within the lipid bilayer, resulting in self-quenching of the fluorophore. Structural damage-induced membrane depolarization leads to the dissipation of the potential and the subsequent release of Disc3(5) into the solution, accompanied by an increase in fluorescence intensity. Figure 5A shows stronger Disc3(5) fluorescence in treated vs. untreated cells, indicating complete membrane depolarization and reduced potential after drug exposure. This observation further confirms that NQP disrupts the structural integrity of bacterial cell membranes.

Extracellular *β*-D-galactosidase activity and ATP content reflect bacterial membrane permeability and are used to evaluate antimicrobial effects on membrane integrity. In our study, NQP treatment increased extracellular β-D-galactosidase concentration and ATP content in AB, suggesting bacterial membrane disruption and enhanced permeability. We also validated the aforementioned conclusion through a PI staining assay. The results indicated that PI was capable of penetrating more extensively into compromised cell membranes, suggesting impairment of membrane integrity.

SEM and TEM provide direct visualization of the morphological changes in bacteria following drug treatment. SEM and TEM analyses revealed that after NQP treatment, AB underwent notable morphological alterations. Specifically, the bacterial cells lost their fullness and exhibited apparent wrinkles, the cell wall and membrane edges showed signs of ablation, and vacuoles emerged within the bacterial body.

### 4.4 Oxidative stress damage

The above results indicated that NQP caused damage to the cell structure of AB and increased the permeability of its cell membrane, potentially disrupting various physiological processes and ultimately resulting in bacterial cell death. Of course, it is plausible that other mechanisms may be involved. We have also conducted a series of experiments related to ROS. When SOD activity is insufficient or NADH levels are elevated, it triggers excessive ROS production. This leads to the accumulation of H₂O₂ and subsequent increase in toxic lipid peroxidation products like MDA, ultimately inducing cellular damage or apoptosis (Belenky et al., 2015). The findings from these experiments revealed that NQP treatment enhanced NADH levels, thereby stimulating oxidative phosphorylation. This subsequently led to a significant increase in hydrogen peroxide and superoxide ROS levels, which caused detrimental effects on bacterial cellular components. Notably, the level of SOD secreted by the bacteria did not increase significantly to counteract this damage, leading to heightened intracellular ROS levels and accelerated bacterial cell death. Furthermore, NQP induced lipid peroxidation in AB, resulting in an increase in MDA content and compromising membrane integrity and permeability.

### 4.5 Transcriptomics analysis

Transcriptomics provides a comprehensive analysis of the changes in gene expression across the entire transcriptome of AB in response to drug intervention, enabling a deeper and more systematic elucidation of the drug’s mechanism of action (Gao and Ma, 2022). In comparison to the control group, a total of 498 genes were found to be differentially expressed in AB following NQP intervention. Further analysis of these genes indicated that multiple metabolic pathways and biological processes, such as oxidative phosphorylation, the TCA cycle, and amino acid metabolism, were affected in AB. Additionally, our study revealed a 3-fold increase in the expression levels of the status genes ndh, aceE and others. Thus NQP accelerated the TCA cycle and oxidative phosphorylation, leading to excessive superoxide production and activation of the ROS-mediated bactericidal process. This, in turn, caused DNA damage and intracellular lipid peroxidation, ultimately resulting in bacterial death.

RT-qPCR results showed that the upregulation of *glmU*, *galE*, *trpS*, and *lolA* gene expression enhances bacterial resistance to environmental stresses. Conversely, the downregulation of *PaaA*, *UreC*, and *gltS* disrupts key metabolic pathways, ultimately leading to bacterial cell death. Notably, *KapA*, a critical regulator of the two-component signal transduction system, exhibits reduced expression, which impairs bacterial stress response and significantly diminishes pathogenicity and antibiotic resistance. In addition, the RT-qPCR results were consistent with RNA sequencing, confirming the reliability of the sequencing data.

### 4.6 Molecular structure analysis

By analyzing the molecular structure of NQP, we can investigate the interplay between its structural features and its antimicrobial mechanism. The quinoline moiety present in NQP has been extensively utilized in drug design and discovery, attributed to its extensive range of biological activities (Yadav and Shah, 2021). Quinolines exhibit a variety of pharmacological properties, including antimalarial, anticancer, antihypertensive, anti-inflammatory, antibacterial, and antiviral activities (Elebiju et al., 2022; Musiol, 2017). Currently, several marketed drugs, notably ofloxacin, norfloxacin, and ciprofloxacin, approved for the treatment of various bacterial infections, incorporate quinoline scaffolds as essential structural elements (Behera et al., 2021; Matada et al., 2021). Thus, the quinoline moiety in NQP offers the potential for exhibiting antimicrobial activity. Of course, it is important to note that reports on the antimicrobial activity, particularly against AB, of 4-aminoquinoline analogs are relatively scarce. Hu et al. reported that a quinoline derivative, HT61, disrupts the membrane potential of methicillin-sensitive *Staphylococcus aureus* (MSSA) bacterial cells by acting on their plasma membrane, leading to the release of cellular contents. Furthermore, they also identified the depolarizing effect of HT61 on MSSA bacterial membranes through the use of Disc3(5) (Hu et al., 2010; Hubbard et al., 2017). Their findings and underlying mechanisms are highly similar to those of our research. The phenolic hydroxyl group in NQP is a prevalent functional group found in numerous natural products and synthetic compounds, and it serves a crucial role in imparting antimicrobial activity to these molecules (Lobiuc et al., 2023). Research has demonstrated that phenolic compounds have the capacity to bind directly to bacterial cell membranes, resulting in their damage. The antimicrobial mechanism entails hydroxyl group accumulation in the lipid bilayer, disrupting lipoprotein interactions and boosting membrane permeability. Phenolic compounds can further weaken membrane integrity, alter cell shape, disrupt metabolism, leading to cellular content leakage (Konuk and Ergüden, 2020; Zhang et al., 2019). Furthermore, phenolic compounds have the ability to induce oxidative stress in bacteria, leading to the production of reactive oxygen species (ROS) that can cause damage to bacterial membranes (Davidova et al., 2024). These mechanisms align with our experimental findings and can consequently be applied to NQP as well.

In clinical practice, the coexistence of bacterial and malaria infections is not uncommon, making it highly beneficial to possess a drug that possesses both antibacterial and antimalarial properties (Endo et al., 2021; Wilairatana et al., 2022). NQP presents an innovative molecular scaffold with potential applications in treating antimicrobial infections, while also offering promising prospects for dual-utility.

## 5 Conclusion

In conclusion, this study unveils the broad-spectrum antibacterial activity of NQP for the first time, with a specific focus on its inhibitory effect on clinically isolated drug-resistant AB, along with its underlying mechanism. The antibacterial mechanism of NQP likely involves disrupting bacterial membranes, which increases cell membrane permeability and triggers oxidative stress, ultimately leading to the destabilization of the bacterial internal environment and disruption of normal metabolism. These findings imply that NQP could serve as a promising molecular scaffold for the exploration of novel therapeutic strategies for treating AB infections. Ongoing research is focused on exploring the *in vivo* pharmacodynamics of NQP and its synergistic effects when combined with other antibiotics. *In vivo* efficacy and toxicity studies in animal models are warranted to validate NQP’s therapeutic index, representing a key direction for future research.

## Data Availability

The original contributions presented in the study are publicly available. This data can be found here: [https://ngdc.cncb.ac.cn/gsa/browse/CRA028470].

## Glossary

NQP: naphthoquine Phosphate
MIC: minimum inhibitory concentration
AMR: antimicrobial resistance
CRE: carbapenem-resistant Enterobacteriaceae
CRAB: carbapenem-resistant *Acinetobacter baumannii*
CRPA: carbapenem-resistant *Pseudomonas aeruginosa*
MDR: multidrug resistance
Ca-MHB: cation-adjusted Mueller-Hinton Broth
CLSI: Clinical and Laboratory Standards Institute
CCK-8: cell counting kit-8
MHA: Mueller-Hinton Agar
PI: propidium iodide
ONPG: ortho-nitrophenyl-*β*-galactoside
ROS: reactive oxygen species
DCFH-DA: dichlorofluorescin diacetate
NADH: nicotinamide adenine dinucleotide (reduced form)
NAD +: nicotinamide adenine dinucleotide (oxidized form).
WST-8: water-soluble tetrazolium-8
SOD: superoxide dismutase
T-SOD: total superoxide dismutase
MDA: Malondialdehyde
GO: gene ontology
KEGG: Kyoto encyclopedia of genes and genomes
RT-qPCR: reverse transcription quantitative polymerase chain reaction
RNA-Seq: RNA sequencing
CFU: colony forming units
FICI: fractional inhibitory concentration index
MOI: multiplicity of infection
TCA: tricarboxylic acid
ECOFF: epidemiological cut-off value
ASTM: American Society of Testing Materials
FC: fold change
